# Editorial: Vision, learning, and robotics: AI for plants in the 2020s

**DOI:** 10.3389/fpls.2024.1539626

**Published:** 2024-12-23

**Authors:** Zhenghong Yu, Luca Iocchi, Jeffrey Too Chuan Tan, Huabing Zhou, Changcai Yang, Hao Lu

**Affiliations:** ^1^ Guangdong Polytechnic of Science and Technology, Zhuhai, China; ^2^ Sapienza University of Rome, Rome, Italy; ^3^ Genovasi University College, Petaling Jaya, Selangor, Malaysia; ^4^ Wuhan Institute of Technology, Wuhan, China; ^5^ Fujian Agriculture and Forestry University, Fuzhou, China; ^6^ Huazhong University of Science and Technology, Wuhan, China

**Keywords:** artificial intelligence, machine learning, robotics, precision agriculture, disease detection, sustainable farming

## Introduction

With the growth of the global population and increasing demand for food, agricultural production is under significant pressure. At the same time, climate change and resource constraints exacerbate these challenges, further heightening the need for sustainable agricultural practices. To address these complex issues, the field of plant science is undergoing a technological revolution. The rapid advancement of artificial intelligence (AI), computer vision, and robotics is redefining how plants are studied and agricultural practices are managed. From high-throughput phenotyping to precision agriculture and real-time monitoring, these technologies are dramatically improving efficiency and accuracy, laying a foundation for more resilient and sustainable agricultural systems. This Research Topic brings together pioneering studies to demonstrate how AI is advancing plant science and providing innovative solutions for modern agriculture.

## Research contributions

The articles in this Research Topic showcase innovations across multiple fields. These contributions can be summarized into five key areas, each highlighting significant advancements in the study and application of plant science.

### High-throughput phenotyping and crop monitoring

High-throughput phenotyping is a critical component of precision agriculture. By incorporating advanced deep learning models, researchers have significantly enhanced the efficiency and accuracy of crop phenotyping. For instance, Li et al. proposed a residual network approach based on hyperspectral imaging, enabling rapid identification of corn varieties while adapting to varying growth conditions. This method not only improves prediction accuracy but also demonstrates the potential of hyperspectral data in agriculture. Additionally, the integration of RGB imaging with environmental variables broadens the scope of crop monitoring, driving the adoption of multimodal data fusion in agricultural applications.

### Applications of robotics and automation in agriculture

Agricultural automation is transforming traditional farming practices. Guo et al. developed an autonomous navigation system for a greenhouse electric crawler tractor based on LiDAR, demonstrating its ability to navigate complex environments accurately. This system combines high-precision sensors with AI algorithms, reducing dependence on manual operation and significantly improving operational efficiency. Furthermore, solutions that integrate ground-based robots with drones have been applied to canopy imaging, weed detection, and disease monitoring, opening new avenues for smart farming.

### Plant disease detection and management

Plant disease detection remains a critical area of agricultural research. Zhou et al. developed an improved ShuffleNetV2 model for rapid identification of field crop leaf diseases. This lightweight model maintains high accuracy while reducing computational requirements, making it well-suited for deployment in resource-constrained agricultural environments. Additionally, Ye et al. proposed enhancements to the YOLOv7 model for large-scale tea leaf disease detection in complex environments. The dual-level routing dynamic sparse attention mechanism employed significantly improves detection accuracy, offering robust support for precision agriculture.

### Predicting plant growth and pruning behavior

Using machine learning to predict plant growth and pruning behavior provides new tools for agricultural decision-making. Shu et al. employed machine learning algorithms to predict the resprouting of Platanus × hispanica after pruning. This study not only reveals the relationship between pruning and plant growth but also offers practical guidance for forestry and horticulture. Moreover, multimodal modeling that integrates plant phenotypic data with environmental variables further enhances the accuracy of growth pattern predictions.

### Food safety and quality monitoring of agricultural products

Improving food safety and quality is a primary goal of modern agricultural research. Afsharpour et al. proposed a robust deep learning method for detecting fruit decay and identifying plants. By integrating advanced image processing and classification algorithms, this method enables rapid identification of decayed fruit, improving efficiency and safety in food processing. Similarly, Kim et al. developed a machine vision-based weight prediction system for butterhead lettuce, providing an effective quality control tool for industrial agriculture.

## Key trends and challenges

This Research Topic highlights several important trends while reflecting on persistent challenges in the field. Firstly, lightweight AI models for on-site deployment are becoming increasingly mainstream. These models maintain high accuracy despite limited computational resources, as demonstrated by Ye et al. Secondly, the rise of multimodal data fusion offers more comprehensive insights for phenotyping and health analysis, exemplified by the integration of RGB imaging and hyperspectral data by Li et al. However, the field continues to face challenges such as data scarcity, high equipment costs, and the complexity of model deployment. Addressing these challenges will require interdisciplinary collaboration, open-access datasets, and innovative engineering solutions.

## Future directions

To advance plant science and achieve sustainable agriculture, future research should focus on the following directions: (1) Developing open-access datasets and affordable hardware to lower the barriers to AI adoption; (2) Optimizing lightweight models to enhance their robustness and scalability for smallholder farms and diverse agricultural environments; (3) Integrating satellite imagery, drones, and ground-based sensors to create a multi-layered crop monitoring system; (4) Exploring the long-term impacts of robotics and AI on agricultural ecosystems, particularly in terms of environmental sustainability and economic equity. These directions will provide new momentum for achieving precision agriculture.

## Conclusion

This Research Topic demonstrates how artificial intelligence, machine learning, and robotics can address critical challenges in modern agriculture by enhancing efficiency and sustainability. The studies not only reveal diverse applications of these technologies in plant science but also lay a foundation for future agricultural innovations. As technology continues to evolve, these breakthroughs will offer new solutions for global food security and ecological conservation.

